# Prospecting Peptides Isolated From Black Soldier Fly (Diptera: Stratiomyidae) With Antimicrobial Activity Against *Helicobacter pylori* (Campylobacterales: Helicobacteraceae)

**DOI:** 10.1093/jisesa/iez120

**Published:** 2019-12-22

**Authors:** Daniela Alvarez, Kevin A Wilkinson, Michel Treilhou, Nathan Téné, Denis Castillo, Michel Sauvain

**Affiliations:** 1 Laboratorio Mixto Internacional Andino Amazónico de Química de la Vida LMI-LAVi, Laboratorios de Investigación y Desarrollo LID, Universidad Peruana Cayetano Heredia, Urb Ingeniería, Lima, Peru; 2 Universidad Privada Antenor Orrego (UPAO), Trujillo, La Libertad, Peru; 3 Equipe BTSB-EA 7417, Université de Toulouse, Institut National Universitaire Jean-François Champollion, Place de Verdun, Albi, France; 4 Institut de Recherche pour le Développement (IRD), Université Paul Sabatier, Toulouse, France

**Keywords:** *Hermetia illucens*, bioprospecting, *Helicobacter pylori*, antimicrobial peptides

## Abstract

*Helicobacter pylori* (Marshall & Goodwin) is a widespread human pathogen that is acquiring resistance to the antibiotics used to treat it. This increasing resistance necessitates a continued search for new antibiotics. An antibiotic source that shows promise is animals whose immune systems must adapt to living in bacteria-laden conditions by producing antibacterial peptides or small molecules. Among these animals is the black soldier fly (BSF; *Hermetia illucens* Linnaeus), a Diptera that colonizes decomposing organic matter. In order to find anti-*H. pylori* peptides in BSF, larvae were challenged with *Escherichia coli* (Enterobacteriales: Enterobacteriaceae). Small peptides were extracted from hemolymph and purified using solid-phase extraction, molecular weight cutoff filtration and two rounds of preparative high performance liquid chromatography (HPLC). The anti-*H. pylori* fraction was followed through the purification process using the inhibition zone assay in brain-heart infusion agar, while peptides from uninoculated larvae had no activity. The inhibition halo of the active sample was comparable to the action of metronidazole in the inhibition zone assay. The purified sample contained four peptides with average masses of approximately 4.2 kDa that eluted together when analyzed by HPLC-mass spectrometry. The peptides likely have similar sequences, activity, and properties. Therefore, BSF produces inducible antibacterial peptides that have in vitro activity against *H. pylori*, which highlights BSF’s position as an important target for further bioprospecting.

*Helicobacter pylori* is a Gram-negative pathogenic bacterium that infects about half of the world’s population (Zamani et al. 2018) and is a causative agent of chronic progressive gastric inflammation, peptic ulcers, gastric adenocarcinoma, and mucosa-associated lymphoid tissue lymphoma (Peek and Crabtree 2006, Wroblewski et al. 2010). Antibiotic therapy has proven effective in eradicating *H. pylori*, but the pathogen is developing resistance to these medicines which necessitates the constant search for new antibiotics. Chemists have had success in bioprospecting for antibiotics in locations with high bacterial loads, from animals living in close contact with bacteria (Akbar et al. 2019) to less studied sources like the deep-sea environment (Tortorella et al. 2018).

An appealing target for bioprospecting is the saprophagous maggot of the black soldier fly (BSF; *Hermetia illucens* Linnaeus). This maggot is a warm-climate, nonpest insect that quickly colonizes decomposing matter (Sheppard et al. 2002). These characteristics suggest it has a potent immune system and can produce antimicrobial substances like peptides (Park et al. 2014, Choi et al. 2018) against a broad spectrum of bacteria (Bahar and Ren 2013, Elhag et al. 2017). An advantage of using peptides as antimicrobials is that they are effective against multidrug resistant bacteria (Chung and Khanum 2017).

A recent study that investigated the effect of diet on the BSF immunity-related transcriptome identified more than 50 genes encoding putative antimicrobial peptides (AMPs) (Vogel et al. 2018). At present only two AMPs have been isolated and characterized from BSF larvae: one displays activity against Gram-positive bacteria (Park et al. 2015), the other acts against Gram-negative bacteria like *Escherichia coli*, *Enterobacter aerogenes* Hormaeche & Edwards (Enterobacterales: Enterobacteriaceae), and *Pseudomonas aeruginosa* Migula (Pseudomonadales: Pseudomonadaceae) (Park and Yoe 2017). Considering the need for new anti-*H. pylori* compounds and that BSF seems to be a good source for novel antibacterials, we prospect for and characterize inducible AMPs that inhibit *H. pylori* using bioassay-directed isolation and mass spectrometry.

## Materials and Methods

### Insects and Inoculation

BSF larvae were grown and maintained under a lighting schedule of 12 h light and 12 h dark at 28 ± 2°C and at 70% relative humidity. An actively growing overnight culture of nonpathogen *E. coli* (ATCC 25922, Manassas, VA; 1 × 10^3^ cells/µl, 10 µl) was injected into the hemolymph of lots of approximately 750 rinsed and dried fourth instar larvae using a tuberculin needle. Cell count was confirmed using a hemocytometer. Inoculated larvae were incubated for an additional 36 h. The experimental control consisted of untreated BSF larvae.

### Hemolymph Extraction

Hemolymph was collected following an established protocol (Hetru and Bulet 1997). In brief, larvae abdomens were sectioned with a scalpel, hemolymph was collected with a tuberculin syringe and deposited in precooled tubes containing aprotinin and phenylthiourea (10 µg/ml and 1 µg/ml respectively). Hemolymph was centrifuged at 15,000 × *g* at 4°C for 3 min. The supernatant was acidified with 0.1% aqueous trifluoroacetic acid (TFA_(aq)_) in an ice-cold water bath and vortexed and centrifuged again (15,000 × *g*, 4°C, 30 min).

### Solid-Phase Extraction

**Fig. 1.**
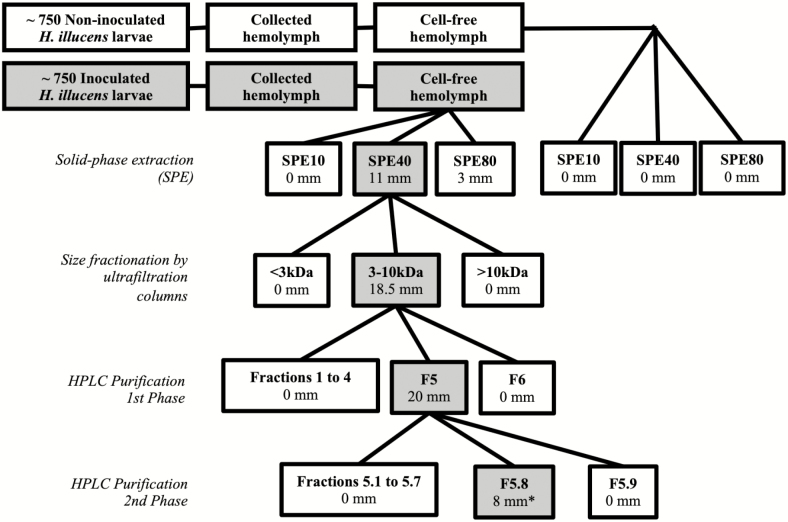
Schematic of biodirected purification of anti-*H. pylori* AMPs. Shaded rectangles indicate anti-*H. pylori* activity is present in the fraction. Distances underneath fraction names report the diameter of the inhibition halo during the inhibition zone assay using 5 µg of sample. (*), 0.63 µg were used for the assay.

Chromabond Sep-Pak C18 cartridges (Macherey-Nagel, Düren, Germany) were equilibrated with methanol and 0.05% TFA_(aq)_. Hemolymph supernate (0.5 ml) was loaded onto the cartridge. AMPs were eluted using three 6 ml aliquots each of 10, 40, and 80% acetonitrile (ACN) with balance of 0.05% TFA_(aq)_, Fig. 1. The three fractions were concentrated by removing solvent in a rotary evaporator and by lyophilization. Fractions were kept at −20°C until use. Relevant peptide concentrations were measured using the Non-Interfering Protein Assay Kit (Merck-Millipore, Darmstadt, Germany; C/N 488250).

### Inhibition Zone Assay

Anti-*H. pylori* (ATCC 43504) activity was evaluated as described previously (Park et al. 2013) but with modifications based on *H. pylori* requirements. Plates containing brain-heart infusion supplemented with 0.8% agar, 10% bovine fetal serum, 2% Isovitalex, 0.025% amphotericin, 0.4% triphenyltetrazolium chloride, and 1 × 10^6^*H. pylori* cells/ml from an actively growing overnight culture were prepared. Cell count was confirmed using a hemocytometer, viability was confirmed by observing cell motility. Test samples were loaded into wells (3 mm) punched out of the agar. The diameter of the inhibition zone was measured after incubation (37°C, 72 h, 10% CO_2_, 5% O_2_, 85% N_2_).

### Size Fractionation by Ultrafiltration Columns

The 40% ACN fraction (2 ml, 1 mg) was loaded into Amicon Ultra-2 (Merck-Millipore, Darmstadt, Germany) NMWL 10 kDa filters and centrifuged at 4,000 × *g* at 4°C for 30 min. The filtrate was then loaded into 3 kDa filters and centrifuged at 4,000 × *g* at 4°C for 1 h; residues and filtrates were tested for activity.

### Preparative HPLC of the Active Fractions

Peptides were eluted from an Aeris Peptides XB-C18, 150 × 2.1 mm; 2.6 µm, 100 Å HPLC column (Phenomenex, Torrance, CA) on a gradient at a flow of 0.5 ml/min from 10 to 45% ACN in 0.1% TFA_(aq)_ over 45 min using an HPLC Dionex Ultimate 3000 UHPLC system (Thermo Scientific, Waltham, MA). Six fractions were collected (Fig. 1) by monitoring absorbance at 215 nm. Fractions were concentrated using a rotary evaporator. The active fraction was subjected to an additional purification step using a modified gradient from 25 to 40% ACN in 0.1 TFA_(aq)_ over 45 min, where an additional nine fractions were collected.

### Sample Analysis by Mass Spectrometry

Intermediately purified peptide samples were separated and analyzed using a UPLC Acquity coupled to a Xevo G2-XS qTOF (Waters, Milford, MA) by injection on a UPLC BEH C18 (2.1 × 50 mm, 1.7 µm) column and eluted using 0.1% aqueous formic acid and ACN/0.5% formic acid on a gradient. Sequence, mass, and disulfide analyses (Touchard et al. 2015) of the final purified product were carried out on an LTQ-Velos Pro Orbitrap (Thermo Scientific, Danforth Plant Science Center, St. Louis, MO); the masses were confirmed on the Xevo G2-XS qTOF.

## Results

### 

Figure 1 tracks the purification of BSF AMPs and reports anti-*H. pylori* activity measured at each step. The first inhibition zone assay indicated that the 10% ACN fraction had no activity, while the 40 and 80% fractions had inhibition halos of 11 mm and 3 mm (5 µg/well). Noninoculated fractions did not have anti-*H. pylori* activity. The 40% fraction (F40) had a recovery of 11 mg of protein and was selected for size-exclusion filtration to remove proteins that were not in the mass range of other known BSF AMPs. The filtered F40 had a halo of 18.5 mm (5 µg/well) and a recovery of 430 µg.

Filtered F40 was then analyzed using mass spectrometry, resulting in a catalogue of over 93 unique cationic, multiply charged peptides with a mass range of 1.6–7.3 kDa; which made necessary additional purification to find the active peptide(s).

**Fig. 2.**
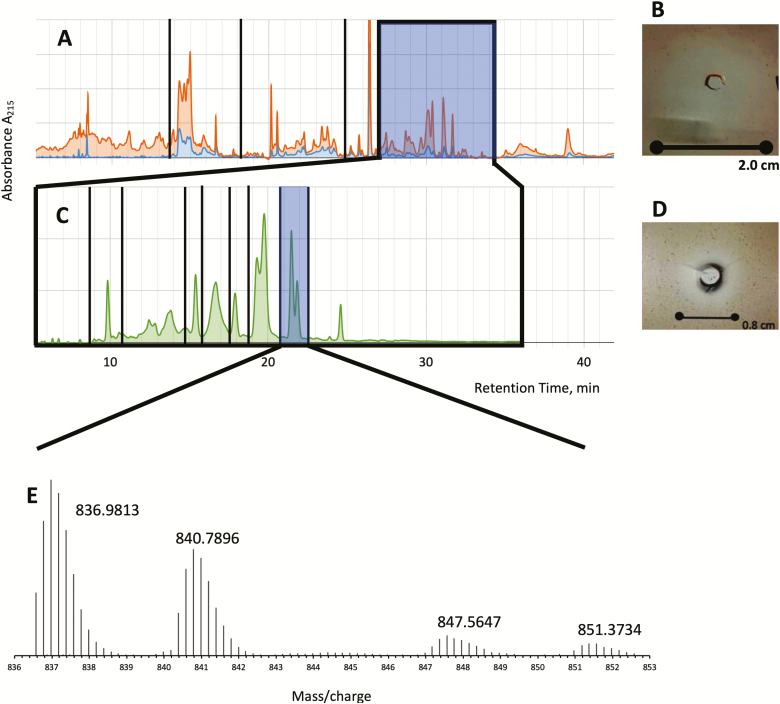
Results of preparative HPLC, inhibition zone, and mass spectrometry analysis of anti-*H. pylori* AMPs. (A) HPLC fractionation of the 3–10 kDa fraction. Baseline-adjusted chromatograms of noninoculated sample (blue) and inoculated sample (orange). Vertical lines indicate different fractions collected. Shaded box reports the active fraction (F5). Inoculated and noninoculated chromatograms were also normalized to each other to reflect relative amounts of sample loading. (B) *H. pylori* inhibition halo of sample F5. The inhibition zone, indicated by absence of colonies and discolored agar, is 2.0 cm wide. (C) Chromatographic separation of F5 (inoculated sample, green). F5.8 with anti-*H. pylori* activity is highlighted. (D) Inhibition zone of the highlighted fraction in (C). (E) MSqTOF spectrum of the active sample in (C). Peak groupings are due to isotope abundances in the peptides. The most abundant mass over charge ratio for each peptide is indicated. The spacing of the peaks, roughly 0.2 Da, indicates a +5 charge on the peptides. +6 and +4 charges were also observed but not shown.

Filtered F40 was separated into six fractions by preparative HPLC (Figs. 1 and 2A). The sample contained many components, some of which were inducible by *E. coli* inoculation (compare orange and blue lines, Fig. 2A). Each fraction was evaluated for anti-*H. pylori* activity, with only fraction 5 (F5, 40 µg recovery) having activity (Fig. 2B, 20 mm, 5 µg/well; Fig. 2A, blue shaded area). Because F5 also had several peptide components of different mass, as exhibited by multiple chromatographic peaks, F5 was subjected to a second round of preparative HPLC (Fig. 2C). The most potent fraction (F5.8, 2 µg recovery) had an inhibition halo of 8 mm (Fig. 2D, 0.63 µg/well; Fig. 2C, blue shaded area).

Fraction 5.8 was then analyzed by UPLC-MSqTOF to determine the exact masses of the peptides in the sample. One chromatographic peak was observed that had masses consistent with peptides. This peak was found to contain four separate peptides with slightly different masses (Table 1 and Fig. 2E). The two most abundant peptides (4180 and 4199 Da) were analyzed for disulfide bridges, and none were found. Tandem mass spectrometry analysis of these two peptides did not provide comprehensive sequence information; however, both tandem mass spectra had prominent peaks at 120.0808 and 157.1337 *m*/*z* and greatly resembled each other in the low-mass range.

**Table 1.** Peptides in F5.8 analyzed by mass spectrometry

Average mass (Da)	Relative abundance	Average charge	*m*/*z*	Charge (+)	Calculated mass (Da)	Relative peak height
4179.8696	14.3	4.7	697.6521	6	4179.8689	14.8
			836.9813	5	4179.8701	105
			1045.9747	4	4179.8697	54.9
4198.9358	8.78	4.8	700.8258	6	4198.9111	10.0
			840.7896	5	4198.9116	64.0
			1050.7534	4	4198.9845	33.0
4232.7873	1.70	4.7	706.4722	6	4232.7895	1.54
			847.5647	5	4232.7871	12.0
			1059.2036	4	4232.7853	7.17
4251.8278	1.00	4.8	709.6456	6	4251.8299	1.00
			851.3734	5	4251.8306	7.12
			1063.9630	4	4251.8229	4.06

## Discussion

We find that BSF larval hemolymph is a source of many AMPs expressed both constitutively and by induction. These peptides range from about 20 to 50 amino acids (1.6–7.3 kDa) and most likely function as part of the immune system to combat infection (Bahar and Ren 2013, Park et al. 2014, Elhag 2017). Through bioassay-directed purification, we were able to identify a set of four coeluting peptides with length of about 40 amino acids that have potent anti-*H. pylori* activity. These peptides are not the most abundant AMPs in hemolymph; combined they represent less than 10% of the putative AMPs in F40. The production of anti-*H. pylori* peptides is enhanced when the larvae are challenged with *E. coli*: expression increases by about 3–5 times based on chromatographic peak height (Fig. 2A). The anti-*H. pylori* activity of the peptides is comparable to metronidazole in the gel diffusion test, where a 21 mm zone of inhibition with metronidazole (5 µg) indicates susceptibility (DeCross et al. 1993, Chaves et al. 1999). This equates to an area of 346 mm^2^. When inhibition areas and amounts added to the plate are compared, F5.8 should surpass these thresholds if sufficient sample was used. Since a 0.63 µg sample inhibited an area of 50 mm^2^, a sample quantity of 5 µg should inhibit about 400 mm^2^ of agar, or a radius of 22 mm.

Some characteristics of the isolated peptides can be inferred from their mass and charge. Given that average charge (Table 1) is a function of the surface area of a protein, the surface area of the peptides should be about 8 Å ^2^. Other common proteins that have similar surface areas tend to have greater masses than the measured AMP masses. Therefore, it is likely that these AMPs have relatively high solvent accessibility when compared with other proteins of similar mass (Kaltashov and Mohimen 2005).

Some sequence information can be obtained from mass spectrometry. First, the sequence of these peptides does not contain cysteine. Second, the tandem mass spectra contain some interpretable sequence information. Tandem mass spectra of the two most abundant peptides contain a prominent peak at *m*/*z* 120.0808 that can be assigned as a phenylalanine immonium ion as well as peaks at 157.1337 and 272.1607 that may be b2 and b3 ions containing N-terminal glycine and valine followed by aspartic acid. In addition, there are several other low-mass peaks in both spectra (at least 17 intense peaks shared between *m*/*z* 100 and 450), indicating that these peptides may have sequence homology. Nonetheless, comprehensive sequence analysis was hindered because sample size was small and because the sample was a mixture of different peptides of similar masses. Revisiting this process with a concentrated pure sample of sufficient size may simplify mass spectra enough to extract interpretable sequence information.

BSF produces a potent antibiotic against *H. pylori* when challenged with *E. coli*. In effect, this assay measured cross reactivity of the peptides, which may indicate that the described AMPs may have a wide spectrum of action (Bahar and Ren 2013, Chung and Khanum 2017). To the best of our knowledge, this is the first time an anti-*H. pylori* peptide has been isolated from insect larvae. Additionally, the mass of the peptides described here compares well with the previously reported mass range of 2.0–4.5 kDa for other anti-*H. pylori* AMPs (Neshani et al. 2019). Given that these peptides coelute during HPLC, and that high sequence homology is expected, it is likely that the purified peptides have very similar antimicrobial, physical and chemical properties.

We hope that these peptides may provide a basis for development of safe and effective medicines to treat *H. pylori* infection. Furthermore, our work underlines the bioprospecting potential of insects in the search for new antibiotic compounds. It is likely that other saprophagous insects will harbor additional AMPs effective against a wide range of important human pathogens, including those that are not normally present in their environment. Potential future investigation could include improving the yield of peptide production in BSF hemolymph, testing resistant strains of *H. pylori*, and additional mass spectrometric analysis to complete the sequence of the peptides.
