# Correction to: Prospecting Peptides Isolated From Black Soldier Fly (Diptera: Stratiomyidae) With Antimicrobial Activity Against *Helicobacter pylori* (Campylobacterales: Helicobacteraceae)

**DOI:** 10.1093/jisesa/ieac053

**Published:** 2022-08-25

This is a correction to: Daniela Alvarez, Kevin A Wilkinson, Michel Treilhou, Nathan Téné, Denis Castillo, Michel Sauvain, Prospecting Peptides Isolated From Black Soldier Fly (Diptera: Stratiomyidae) With Antimicrobial Activity Against *Helicobacter pylori* (Campylobacterales: Helicobacteraceae), *Journal of Insect Science*, Volume 19, Issue 6, November 2019, 17, https://doi.org/10.1093/jisesa/iez120

In the originally published version of this manuscript, the second paragraph of the Discussion incorrectly states that “the surface area of the peptides should be about 8 Å^2^.” There was a mistake in this calculation and the result should read “the surface area of the peptides should be about 3500 Å^2^.”

This detail has been corrected only in this correction notice to preserve the published version of record.

